# Modelling how antimicrobial resistance spreads between wards

**DOI:** 10.7554/eLife.64228

**Published:** 2020-11-26

**Authors:** Tjibbe Donker

**Affiliations:** Institute for Infection Prevention and Hospital Epidemiology, University Medical Center FreiburgFreiburgGermany

**Keywords:** antimicrobial resistance, antibiotic stewardship, infection control, Klebsiella pneumoniae, MRSA, antibiotics, *E. coli*, Other

## Abstract

Moving patients between wards and prescribing high levels of antibiotics increases the spread of bacterial infections that are resistant to treatment in hospitals.

**Related research article** Shapiro JT, Leboucher G, Myard-Dury AF, Girardo P, Luzzati A, Mary M, Sauzon JF, Lafay B, Dauwalder O, Laurent F, Lina G, Chidiac C, Couray-Targe S, Vandenesch F, Flandrois JP, Rasigade JP. 2020. Metapopulation ecology links antibiotic resistance, consumption, and patient transfers in a network of hospital wards. *eLife* **9**:e54795. doi: 10.7554/eLife.54795

Across the world, there are increasing numbers of microbes that are able to survive antibiotic treatment. This antimicrobial resistance (or AMR for short) is reducing the number of drugs available to fight off bacterial infections, sometimes to the extent that even the last line of treatment is no longer effective (Friedman et al., 2016). Many of the deaths associated with AMR occur in hospitals, which are ideal breeding grounds for resistant bacteria due to the high amounts of antibiotics consumed and the frail patient population (de Kraker et al., 2011; Naylor et al., 2019).

AMR is traditionally studied within individual hospital wards, where bacteria are transmitted between patients through contact with healthcare workers. However, wards are not individual entities because patients are routinely transferred between them. Every time a patient is transferred to a new ward, there is the potential that they may bring new antibiotic-resistant bacteria with them. As a result, all hospital wards – and consequently all the hospitals in a country – are connected in a large network that AMR bacteria can easily spread through (Nekkab et al., 2017).

Another driving force behind the spread of AMR is bacterial selection, which happens when microbes that are resistant to antibiotics outcompete and replace those that are more susceptible. In hospitals, it is likely that most bacterial populations already contain some resistant bacteria that have an advantage due to the high volume of antibiotics consumed. However, it is largely unknown how the combination of bacterial selection and the transfer of patients between wards shape the spread of AMR within a hospital network.

Now, in eLife, Jean-Philippe Rasigade and co-workers from Université de Lyon and the Hospices Civils de Lyon – including Julie Teresa Shapiro as first author – report how seven species of bacteria, and their resistant strains, spread across 357 wards of a major hospital organisation in Lyon (Shapiro et al., 2020). To do this, the team adapted a model that is often used in ecology to study populations of animal species that live in, and migrate between, different locations. In the model, the migration of bacterial species was calculated by multiplying the number of bacteria in the original ward by the number of patients moved. Using this model, Shapiro et al. were able to examine how the abundance of bacterial species varied depending on the type of ward, how connected it is, and how many antibiotics patients in the ward consumed (Figure 1).

**Figure 1.**
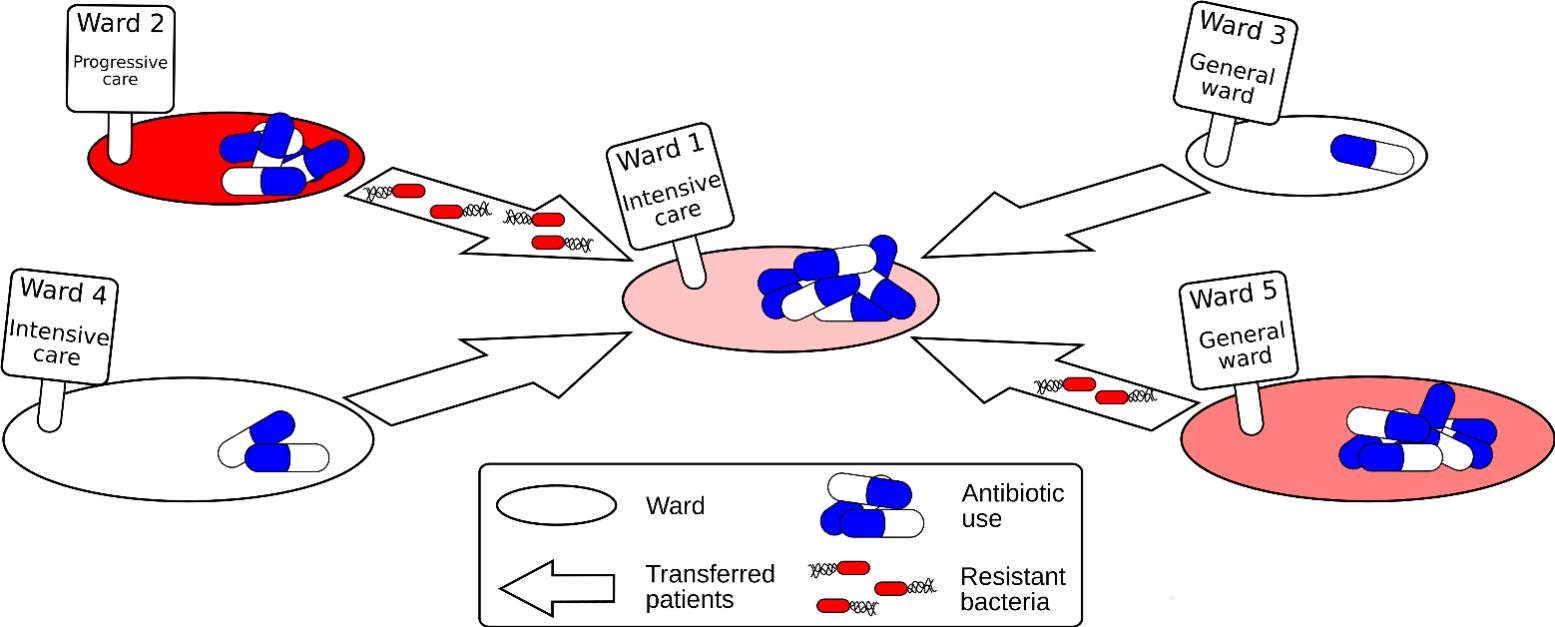
Schematic diagram showing how resistant bacteria spread between hospital wards. Each hospital ward has certain properties, such as its size, the type of ward (e.g. general, intensive and progressive care), and the number of antibiotics it consumes. When patients are moved between wards, resistant bacteria can also be transferred with them. If the level of antibiotic used in the new ward is high, the resistant bacteria may have a selection advantage, causing a rise in antimicrobial resistance.

Shapiro et al. found that both the antibiotics used and the connectivity between wards influenced the number of patients infected with one of the seven studied strains of bacteria. However, these properties affected AMR differently depending on the bacterial species. For instance, two strains of resistant bacteria that are commonly found in hospitals, *Pseudomonas aeruginosa* and *Enterococcus faecium,* were found in higher numbers when more patients were being moved between wards: this increase is likely due to these patients spreading bacteria from different parts of the hospital. However, the spread of other species, such as *Klebsiella pneumoniae,* was more strongly affected by the level of antibiotics used.

This study highlights why the strategies used to control AMR should be specific for the bacterial strain that caused the resistant infection. Many hospitals have implemented antibiotic stewardship programmes, which reduce the selection of resistant bacteria by limiting the number of antibiotics prescribed and administered to patients. Although these programmes control some resistant strains, this strategy may not work for all bacteria. This is because increases in AMR are caused by the amplification of existing resistant strains rather than mutations that allow non-resistant bacteria to become resistant. The best way to reduce the spread of resistant bacteria is therefore to prevent patients from other wards introducing new strains to uninfected areas. This suggests that effectively controlling AMR is going to require studying the multiple wards and hospitals that make up a country’s connected healthcare system.
